# Suberoylanilide hydroxamic acid suppresses axonal damage and neurological dysfunction after subarachnoid hemorrhage via the HDAC1/HSP70/TDP-43 axis

**DOI:** 10.1038/s12276-022-00761-9

**Published:** 2022-05-02

**Authors:** Kui Luo, Zhifei Wang, Kai Zhuang, Shishan Yuan, Fei Liu, Aihua Liu

**Affiliations:** 1 Department of Neurosurgery, The Third Xiangya Hospital, Central South University, 410013 Changsha, China; 2 Medical College, Hunan Normal University, 410000 Changsha, China; 3 Department of Neurosurgery, The Fifth Affiliated Hospital of Sun Yat-Sen University, 519000 Zhuhai, China; 4 Beijing Neurosurgical Institute, Beijing Tiantan Hospital, Capital Medical University, 100070 Beijing, China

**Keywords:** Neuroscience, Molecular biology

## Abstract

Increased focus has been placed on the role of histone deacetylase inhibitors as crucial players in subarachnoid hemorrhage (SAH) progression. Therefore, this study was designed to expand the understanding of SAH by exploring the downstream mechanism of the histone deacetylase inhibitor suberoylanilide hydroxamic acid (SAHA) in SAH. The expression of TDP-43 in patients with SAH and rat models of SAH was measured. Then, western blot analysis, immunofluorescence staining, and transmission electron microscope were used to investigate the in vitro effect of TDP-43 on a neuronal cell model of SAH established by oxyhemoglobin treatment. Immunofluorescence staining and coimmunoprecipitation assays were conducted to explore the relationship among histone deacetylase 1 (HDAC1), heat shock protein 70 (HSP70), and TDP-43. Furthermore, the in vivo effect of HDAC1 on SAH was investigated in rat models of SAH established by endovascular perforation. High expression of TDP-43 in the cerebrospinal fluid of patients with SAH and brain tissues of rat models of SAH was observed, and TDP-43 accumulation in the cytoplasm and the formation of inclusion bodies were responsible for axonal damage, abnormal nuclear membrane morphology, and apoptosis in neurons. TDP-43 degradation was promoted by the HDAC1 inhibitor SAHA *via* the acetylation of HSP70, alleviating SAH, and this effect was verified in vivo in rat models. In conclusion, SAHA relieved axonal damage and neurological dysfunction after SAH *via* the HSP70 acetylation-induced degradation of TDP-43, highlighting a novel therapeutic target for SAH.

## Brain hemorrhage: Enzyme inhibitor limits damage

Analysis of the molecular processes involved in the progression of hemorrhage-induced brain damage has yielded a potential therapeutic target. Fei Liu and Aihua Liu at the Central South University in Changsha, China, and co-workers examined mechanisms behind the extensive brain damage that can be caused by subarachnoid hemorrhage, bleeding in a space between the brain and its surrounding membranes. In experiments on human samples and rat models, the researchers showed that the TDP-43 protein was over-expressed in cerebrospinal fluid after hemorrhage, and that the build-up of this protein aggravated nerve cell damage and dysfunction. TDP-43 is regulated by a pathway involving a potent enzyme involved in regulation of gene expression called HDAC1. Treatment of rat models with an HDAC1 inhibitor degraded TDP-43 and limited the progression of brain damage.

## Introduction

Subarachnoid hemorrhage (SAH) is a lethal and devastating intracranial hemorrhage that is frequently misdiagnosed in the initial stage and increases the risk of mortality and disability^1,2^. SAH, which is mainly induced by aneurysmal rupture and peri-mesencephalic bleeding, is a dangerous disease that is commonly characterized by acute headache, nausea/vomiting and neck pain, with a mortality rate within 24 h of 25% and an overall mortality rate of 50%^3^. Accumulating evidence has suggested that SAH-induced early brain injury is a key factor that affects the prognosis of SAH patients^4^. Although great achievements have been made in understanding the pathogenesis and pathophysiology of SAH, as well as in the treatment and management of SAH, its underlying molecular, cellular, and circulatory dynamics remain largely unclear, and its prognosis is still not satisfactory^5,6^. Thus, in-depth exploration of SAH is needed to improve management.

Suberoylanilide hydroxamic acid (SAHA), also called vorinostat, belongs to the hydroxamic acid class of histone deacetylase (HDAC) inhibitors^7,8^. It was shown that SAHA exerted great clinical utility as a therapeutic intervention after intracerebral hemorrhage^9^. Moreover, HDACs are potent enzymes that posttranslationally modify both histone and nonhistone acetylation sites, thereby affecting many cellular processes, such as the cell cycle and apoptosis^10^. Notably, the exacerbating effects of HDAC4 on SAH can be inhibited by SAHA^11^. HDAC1 inactivation was shown to protect against neuronal death and brain injury^12^. Moreover, it was identified that heat shock protein 70 (HSP70) was a cytosolic substrate of HDAC5 and could be hyperacetylated by HDAC5, which was further associated with the proliferation of hypoxic tumor cells^13^. HSP70 is a well-known therapeutic target for SAH^14^. In addition, HSP70 was shown to inhibit the cytoplasmic accumulation of TDP-43, which is responsible for the formation of insoluble inclusion bodies and is a hallmark of neurodegenerative diseases^15,16^. Moreover, TDP-43 was identified as a prognostic biomarker for SAH^17^. Herein, SAHA is hypothesized to be involved in the development of SAH by interacting with the HDAC1/HSP70/TDP-43 axis.

## Materials and methods

### Ethics statement

The current study was approved by the IRB of The Third Xiangya Hospital of Central South University and was performed in accordance with the Helsinki Declaration. Written informed consent was obtained from each participant. Animal experiments were approved by the Animal Ethics Committee of The Third Xiangya Hospital of Central South University and Central South University and were conducted according to the Guide for the Care and Use of Laboratory Animals published by the US National Institutes of Health.

### Bioinformatics analysis

Aneurysmal SAH-related microarray data (GSE54083) were downloaded from the Gene Expression Omnibus (GEO) database. Because the occurrence of SAH was induced by rupture of an intracranial aneurysm, eight cases of intracranial aneurysm rupture samples and ten normal control samples in GSE54083 were selected, and the differential analysis was conducted using the “limma” package of R language. Differentially expressed genes were identified with a threshold of *P* < 0.05.

### Clinical samples

A total of 15 patients with aneurysmal SAH and 10 patients without SAH (non-SAH patients) from The Third Xiangya Hospital of Central South University were selected for cerebrospinal fluid collection. Cerebrospinal fluid (5 mL) was collected every 6 h for 8 days and then immediately centrifuged in a 10 mL sterile centrifuge tube at 12,000 × *g* for 15 min at 4 °C. The supernatant of the cerebrospinal fluid, which was free from blood cell components, was collected, subpackaged in 1.5 mL centrifuge tubes in equal amounts, and stored at −80 °C for subsequent experiments. Control cerebrospinal fluid and blood samples were collected from age-matched patients who underwent elective surgery that was not associated with the central nervous system.

### Enzyme-linked immunosorbent assay (ELISA)

ELISA was performed to measure the levels of TDP-43 in cerebrospinal fluid based on the instructions of the kit (R&D Systems, Minneapolis, MN, MAB77782, https://www.rndsystems.com/cn).

### Construction of rat models of SAH

A Wistar rat endovascular puncture model of SAH was constructed according to previous research methods^18,19^. A similar operation was performed on control rats, except that the suture was inserted less than 8 mm to avoid ACA perforation. Body temperature was monitored during the operation. During the operation and within 2 h after the operation, heating plates were used to keep the animal’s body temperature at 36.5–37.5 °C. After being anesthetized with 3% sodium pentobarbital, SAH rats were fixed in a stereotaxic apparatus (RWD Life Science, Shenzhen, Guangdong, China), and 15 μL (μg/μL) of lentivirus [short hairpin RNA-negative control (sh-NC), sh-histone deacetylase 1 (sh-HDAC1), overexpression (oe)-NC, and oe-TDP-43] were injected into the rat lateral ventricle (1.0 mm after the bregma, 2.0 mm on the right side, 3.5 mm in-depth) within 10 min at a constant rate (5 μL/min). The needle was maintained for 10 min to prevent blood reflux and then slowly pulled out, after which the scalp was sutured. The success rate of the model was ~85% (68/80, 12 died). Rats were subjected to sham operation or SAH modeling. After successful modeling, the rats were treated with oe-NC, sh-NC, oe-TDP-43, sh-HDAC1, dimethyl sulfoxide (DMSO, 5% in normal saline, injected every day), or SAHA (50 mg/kg, injected every day) or without any injection (*n* = 6 each). No rats died during viral infection or drug treatment.

### Isolation and culture of primary neurons and the construction of cell models of SAH in vitro

Primary neurons were isolated from the brain tissues of fetal rats (embryos aged 16–18 d). The meninges and blood vessels were removed, and the brain tissues were digested with 0.25% trypsin (containing ethylenediaminetetraacetic acid (EDTA)) at 37 °C for 5 min. Then, the tissues were washed three times with phosphate-buffered saline (PBS) to stop the trypsinization. Next, the cells were resuspended on neural basal medium supplemented with 2% B27, 2 mM l-glutamine, 50 U/mL penicillin, and 50 U/mL streptomycin (GIBCO BRL, Grand Island, NY, USA). Half of the medium was renewed every 2 days. An in vitro cell model of SAH was established by stimulating neurons with oxyhemoglobin (OxyHb). The neurons were incubated with OxyHb (20 μM) at 37 °C with 5% CO_2_ for 6 h. Next, the medium was removed, and the neurons were washed with PBS (three times) for subsequent experiments.

### Cell culture and infection

Human embryonic kidney (293T) cells (American Type Culture Collection) were cultured in Dulbecco’s Modified Eagles Medium (Gibco) with 10% (v/v) fetal bovine serum. Primary neurons were cultured in neural basal medium (Gibco) in a cell incubator at 37 °C with 5% CO_2_.

293T cells were cotransfected with the packaging virus and the target vector with LV5-green fluorescent protein (GFP) (lentiviral gene overexpression vector) and pSIH (lentiviral gene silencing vector) for 48 h, followed by supernatant collection. After filtration and centrifugation, viral particles in the supernatant were obtained, and the viral titer was determined. Viruses in the logarithmic growth phase were collected. Cells were treated with OxyHb (treated with 20 μM OxyHb for 24 h), OxyHb + sh-NC (treated with 20 μM OxyHb for 24 h after being transfected with sh-NC for 48 h), OxyHb + sh-HDAC1 (treated with 20 μM OxyHb for 24 h after being transfected with sh-HDAC1 for 48 h), OxyHb + sh-TDP-43 (treated with 20 μM OxyHb for 24 h after being transfected with sh-HDAC1 for 48 h), OxyHb + DMSO (treated with 20 μM OxyHb for 24 h after being transfected with 5% DMSO for 48 h), and OxyHb + SAHA (treated with 20 μM OxyHb for 24 h after being transfected with SAHA for 48 h). sh-NC, sh-HDAC1, oe-NC, and oe-TDP-43 were all synthesized by GenePharma Co., Ltd. (Shanghai, China). Cells were prepared into a 4 × 10^5^ cells/mL cell suspension, seeded in a six-well plate in 2 mL per well, and cultured overnight.

### Garcia behavioral score

Neurological status was evaluated 72 h after SAH modeling. The modified Garcia test included vibrissae touch, trunk touch, spontaneous movement, spontaneous movement of limbs, forelimb extension, and climbing ability (maximum score = 18). Neurological evaluations were performed by an investigator who was blinded to the experimental conditions.

### Rotarod experiment

This test was used to evaluate the exercise ability of rats by rotating a rod cylinder (ZH-300B, Zhenghua Biologic Apparatus Facilities Co., Ltd., Anhui, China)^20^. All rats were trained 3 d before modeling and were tested 1 d before SAH modeling and 1, 3, 7, and 14 d after SAH modeling.

### Morris water maze

The Morris water maze test was carried out as previously described in a circular tank with a diameter of 180 cm and a depth of 50 cm^21^. Starting from the 18th d after modeling, all rats were trained for 4 d, three times per day. Each training session lasted for 1 min, with 5 min intervals between every two sessions. The test was performed from the 22nd to the 26th d after SAH modeling.

### Brain water content analysis

Brain water content was measured by the wet/dry method^22^. In short, rats were deeply anesthetized with pentobarbital sodium 72 h after SAH modeling and euthanized. An electronic analytical balance (Sartorius BS 210 S, Göttingen, Germany) was used to weigh each part.

### Immunohistochemical (IHC) staining

Brain section (4 μm in thickness) were prepared and treated with an EDTA buffer solution (0.05 mol/L Tris, 0.001 mol/L EDTA, pH 8.5), followed by antigen retrieval. Endogenous peroxidase activity was inactivated with 0.3% H_2_O_2_ for 10 min. After being blocked with 5% bovine serum albumin (BSA) for 20 min, the sections were incubated with primary antibodies against amyloid precursor protein (APP, ab101492, 1:100, Abcam, Cambridge, UK) overnight at 4 °C and biotinylated goat anti-mouse immunoglobulin G (IgG) secondary antibodies (ab190475, 1:500, Abcam) for 20 min. Finally, the sections were stained with 3,3-diaminobenzidine and counterstained with hematoxylin. Images were obtained with a microscope (Leica-DM2500, Wetzlar, Germany). Two independent pathologists evaluated the sections. The rate of positive cells for each sample was counted according to the APP staining intensity.

### Immunofluorescence and terminal deoxyribonucleotidyl transferase-mediated 2’-deoxyuridine 5’-triphosphate nick end labeling (TUNEL) staining

Sections were washed in PBS and blocked in 10% BSA at 25 °C for 1 h. Then, the sections were incubated with the following primary antibodies (all from Abcam) at 4 °C for 12–16 h: rabbit anti-HDAC1 (ab109411, 1:100), mouse anti-HSP70 (ab2787, 1:100; Abcam), rabbit anti-TDP-43 (ab109535, 1:100), rabbit anti-Iba1 (ab109535, 1:500), rabbit anti-GFAP (ab33922, 1:500), and rabbit anti-NeuN (ab177487, 1:300). Then, the sections were incubated with Alexa Fluor 647 donkey anti-goat IgG (Thermo Fisher Scientific, Waltham, MA, USA), Alexa Fluor 488 donkey anti-mouse IgG (Thermo Fisher Scientific), and CY3 donkey anti-rabbit IgG (Jackson ImmunoResearch Laboratories, West Grove, PA, USA) at 25 °C for 1 h. Next, the sections were stained with 4’,6-diamidino-2-phenylindole (10 μg/mL, Sigma–Aldrich, St. Louis, MO, USA) at 25 °C for 15 min. Transferase-mediated TUNEL (Cell Death Detection Kit, Roche, Basel, Switzerland) costaining with rabbit anti-NeuN (1:200, Abcam) and neuronal apoptosis were assayed. The sections were observed with an Olympus BX51 fluorescence microscope (Olympus, Tokyo, Japan) or a laser scanning confocal microscope (FV500, Olympus). The image control parameters were set by the control group, and all other parameters remained the same for image capture. Five hematoma border regions were selected in each part for analysis, and four parts were selected for each animal (*n* = 6). ImageJ (National Institutes of Health, Bethesda, Maryland, USA) was used to count the total number of TUNEL and NeuN double-positive cells in five areas near the injury area.

### Western blot analysis

Western blot analysis was performed with following primary antibodies: Dynactin (ab52611, 1:1000, Abcam), neurofilament light chain (NFL) (ab223343, 1:1000, Abcam), ApoE (#13366, 1:1000, Cell signaling Technology, Danvers, MA), TDP-43 (ab109535, 1:1000, Abcam), Acetyl-K (#9441, 1:2000, Abcam), glyceraldehyde-3-phosphate dehydrogenase (GAPDH, ab8245, 1:1000, Abcam), and Lamin A (ab133256, 1:1000, Abcam), as well as horseradish peroxidase-labeled goat anti-rabbit IgG (1:20,000, ab205718, Abcam). Then, a developing solution (NCI4106, Pierce, Rockford, IL, USA) was used for development. ImageJ 1.48 u software (Bio-Rad, Hercules, CA, USA) was used to perform quantitative protein analysis (internal reference: GAPDH).

### Reverse transcription–quantitative polymerase chain reaction (RT–qPCR)

Cells were lysed using a TRIzol kit (Invitrogen, Carlsbad, CA, USA), and total RNA was extracted from cell and tissue samples. RNA quality and concentration were measured by UV–Vis spectrophotometry (ND-1000, Thermo Fisher Scientific). For mRNA analysis, an RT kit (RR047A, Takara, Tokyo, Japan) was used to perform RT to obtain cDNA. Subsequently, cDNA was used as a template, and the SYBR^®^ Premix Ex Taq^TM^ II kit (Perfect Real Time, DRR081, Takara) was used to perform fluorescent qPCR. The samples were subjected to RT–qPCR in a real-time fluorescent qPCR instrument (ABI 7500, ABI, Foster City, CA, USA). The 2−^ΔΔ^CT method was used to quantify expression, and GAPDH was used as the internal reference. The primers are shown in Supplementary Table 1.

### Nuclear and cytoplasmic separation experiment

A NE-PER™ Nuclear and Cytoplasm Extraction Kit (78833, Thermo Fisher Scientific, https://www.thermofisher.cn/) was used to separate the nucleus and cytoplasm in each group of cells, as well as to separate nuclear and cytoplasmic proteins. Then, western blot analysis was performed to measure the expression of TDP-43 (ab109535, 1:1000, Abcam). GAPDH (ab8245, 1:1000, Abcam) was used as the internal reference for cytoplasmic proteins, and Lamin A (ab133256, 1:1000, Abcam) was used for nucleoproteins. The ratio of the gray value of TDP-43 to the gray value of each control protein was used for quantitative analysis by ImageJ 1.48 u software (Bio-Rad, Hercules, CA, USA).

### Determination of malondialdehyde (MDA) levels and superoxide dismutase (SOD) activity

After 72 h, the brain was perfused with PBS, and then the ipsilateral cortex was homogenized to measure MDA levels and the activity of SOD. The MDA level was measured by reacting MDA and thiobarbituric acid under acidic conditions (high temperature). SOD activity was measured by the WST-1 method.

### Fluoro-Jade C (FJC) staining

Brain sections were deparaffinized, dehydrated, incubated in a 0.06% potassium permanganate solution (Sigma–Aldrich) for 10 min, and reacted in FJC working solution (0.1% acetic acid) for 20 min. A fluorescence microscope (Olympus) was used to observe FJC-positive cells, and the number was counted by technicians who were blinded to experimental conditions.

### Dihydroethidium (DHE) staining

DHE staining was carried out to measure superoxide anions, which indicate the level of oxidative stress in tissues^23^. The ventral side of the left hemisphere was examined with the help of a laser scanning confocal microscope (A1 Si, Nikon, Tokyo, Japan). The representative image was obtained from a Section 2 mm posterior to the bregma.

### Coimmunoprecipitation (Co-IP) assay

Equal amounts of cell lysates (1500 µg) were immunoprecipitated with 1 μg of HSP70, HDAC1, TDP-43, or acetyl-K antibodies and 50 μL protein A/G agarose. The immunoprecipitate was washed twice with 10 mM HEPES (pH 7.9), 1 mM EDTA, 150 mM NaCl, and 1% Nonidet P-40 and boiled for 10 min. Then, the precipitated proteins were eluted with 30 μL of sodium dodecyl sulfate (SDS)–PAGE buffer. The eluted proteins were separated by 8% SDS–PAGE, transferred to a nitrocellulose membrane, and examined with the corresponding antibodies.

### Flow cytometry

After 48 h of transfection, the cells were detached with EDTA-free 0.25% trypsin (YB15050057, YuBo Biotech Co., Ltd., Shanghai, China), collected in a flow tube, and centrifuged, and the supernatant was discarded. According to the directions of the Annexin-V-fluorescein isothiocyanate (Annexin-V-FITC) cell apoptosis detection kit (K201-100, BioVision, Milpitas, CA, USA), the Annexin-V-FITC/PI dye solution was prepared. Then, 1 × 10^6^ cells were resuspended in 100 μL of dye solution, incubated at room temperature for 15 min, and 1 mL HEPES buffer solution was added (PB180325, Procell, Wuhan, Hubei, China), followed by cell apoptosis detection.

### Measurement of neurite length

ImageJ software was used to quantify neurite length. Images of cells labeled with anti-GFP and anti-neurofilament antibodies were captured by an Axiovert camera (images were processed using Volocity software). The Neuron J ImageJ plug-in was used to measure the lengths of neurites. Approximately 150 cells under each experimental condition were analyzed by three independent researchers.

### Transmission electron microscopy (TEM)

The grid was checked at 80 KV on a Philips CM120 electron microscope with a low-magnification image (X4, X800 or X7000) of the white matter area containing the axon tract, as well as a high-magnification image (X15000 or X20000) of axons and other subcellular elements that were captured. From the examined samples, axons with myelin sheaths and axon organelles conformed to the ultrastructural standards. TEM images were captured with a charge-coupled device camera (Gatan, Pleasanton, CA, USA) and processed with Digital Micrograph software (Gatan).

### Statistical analysis

SPSS 21.0 statistical software (IBM Corp., Armonk, NY, USA) was used for statistical analysis. Measurement data are displayed as the mean ± standard deviation. First, the normality and homogeneity of variance were tested. Data with a normal distribution and even variance between two groups were analyzed by unpaired *t* tests, while data comparisons among multiple groups were performed using one-way analysis of variance (ANOVA). Comparisons among data at different time points were performed by repeated-measures ANOVA with Tukey’s post-hoc test. A value of *P* < 0.05 was considered statistically significant.

## Results

### TDP-43 was elevated in SAH patients and a rat model of SAH

**Fig. 1:**
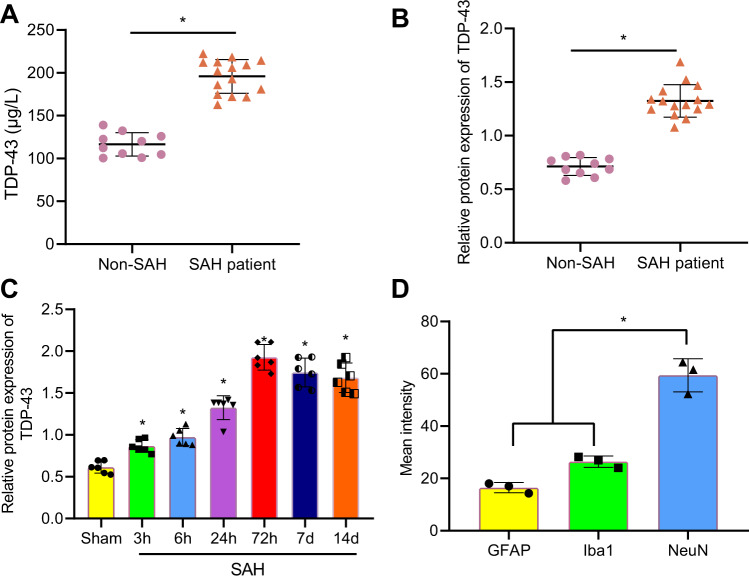
TDP-43 is upregulated and localized in the neurons of SAH patients and a rat model of SAH. **a** The levels of TDP-43 in the cerebrospinal fluid of SAH patients (*n* = 15) and non-SAH patients (*n* = 10) were determined by ELISA. **b** The protein level of TDP-43 in the cerebrospinal fluid of SAH patients (*n* = 15) and non-SAH patients (*n* = 10) was determined by western blot analysis. **c** The protein level of TDP-43 in the brain tissues of rats at 3 h, 6 h, 24 h, 72 h, 7 d, and 14 d after SAH modeling was determined by western blot analysis, *P < 0.05 compared to sham-operated rats. **d** TDP-43 expression and localization in different neurons was examined by immunofluorescence staining (neuronal marker: NeuN, astrocyte marker: GFAP, microglial marker: Iba1). *P < 0.05, *n* = 6. The experiment was repeated three times independently.

To explore the mechanism of TDP-43 in SAH, cerebrospinal fluid was collected from 10 non-SAH patients and 15 patients with aneurysmal SAH. We found that the expression of TDP-43 in the cerebrospinal fluid of SAH patients was much higher than that in the CSF of non-SAH patients (Fig. 1a, b).

Then, a rat model of SAH were constructed by the endovascular puncture. In model rats, we found severe cerebral hemorrhage, increased brain water content (Supplementary Fig. 1a), severe neurological damage (Supplementary Fig. 1b), impaired motor function (Supplementary Fig. 1c), and learning and memory impairment (Supplementary Fig. 1d). The number of DHE-positive cells increased dramatically, SOD activity decreased, and MDA levels increased in rats with SAH (Supplementary Fig. 1e, f). TUNEL and FJC staining demonstrated that the number of apoptotic neuronal cells and neuronal cell degeneration notably increased in rats with SAH (Supplementary Fig. 1g, h). IHC staining of APP showed that there was a large number of positive local accumulations in the white matter area of rats with SAH (Supplementary Fig. 1i). TEM revealed that there were a variety of ultrastructural features of myelinated and unmyelinated axonal damage at the cerebral infarcts and hairy infarcts of rats with SAH, including axon enlargement, nerve fiber compaction, and organelle aggregation (Supplementary Fig. 1j). Therefore, the rat model of SAH was successfully constructed and featured cognitive impairment and axonal injury.

The expression of TDP-43 in the brain tissues of SAH rats at 3 h, 6 h, 24 h, 72 h, 7 d, and 14 d after SAH modeling was examined, and the results showed that TDP-43 increased notably after SAH modeling, and the highest expression was observed at 72 h (Fig. 1c). Immunofluorescence analysis revealed that the fluorescence intensity of TDP-43 in neurons was much higher than that in microglia or astrocytes (Fig. 1d). In summary, TDP-43 was mainly located in neurons and was highly expressed in the cerebrospinal fluid of SAH patients and a rat model of SAH.

### TDP-43 overexpression promoted cognitive impairment and axonal damage in rats with SAH

**Fig. 2:**
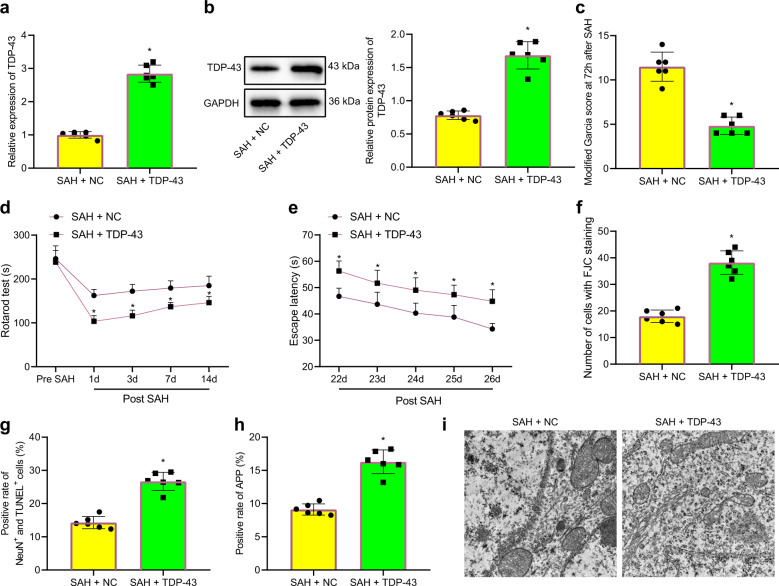
TDP-43 induces neuronal dysfunction, axonal damage, and cell apoptosis in rats with SAH. **a** TDP-43 mRNA levels in rats after different infections were determined by RT–qPCR. **b** TDP-43 protein levels in rats after different infections were determined by western blot analysis. **c** Nerve damage in rats after different infections were evaluated by the modified Garcia behavior score. **d** The movement of rats after different infections was determined by the rotarod test. **e** Memory impairment in rats after different infections was examined by the Morris water maze test. **f** The degeneration of cortical neurons in rats after different infections was detected by FJC staining. **g** Neuronal apoptosis was examined by TUNEL and NeuN double staining. **h** APP expression in rats after different infections was determined by IHC staining. **i** Axonal damage was observed by TEM. *P < 0.05 compared with SAH rats infected with oe-NC, *n* = 6. The experiment was repeated three times independently.

The lateral ventricle was injected with TDP-43-overexpressing lentivirus to determine the mechanism by which TDP-43 regulated cognitive impairment in SAH. The level of TDP-43 mRNA in the brain tissues of rats 72 h after SAH modeling was determined by RT–qPCR. The results demonstrated that the expression of TDP-43 in rats with SAH that were infected with oe-TDP-43 was sharply increased compared with that in rats with SAH that were injected with oe-NC (Fig. 2a). Three rats were randomly selected, and the level of TDP-43 protein in brain tissues was analyzed by western blotting. The expression of TDP-43 in rats after infection with oe-TDP-43 was notably increased (Fig. 2b). Cognitive tests revealed that oe-TDP-43 increased nerve function damage, reduced exercise capacity, impaired memory function, and increased total travel distance in the water maze in rats with SAH (Fig. 2c–e). Rats that were treated with oe-TDP-43 exhibited an increased number of FJC-positive cells and NeuN^+^TUNEL^+^ cells (Fig. 2f, g), indicating that neuronal injury was intensified. In addition, the number of APP-positive cells in rats infected with oe-TDP-43 was elevated (Fig. 2h). Compared with that in rats infected with oe-NC, the number of swollen and dystrophic axons in rats infected with oe-TDP-43 increased dramatically (Fig. 2i), indicating that axon damage was enhanced. In summary, TDP-43 overexpression further deteriorated cognitive dysfunction and axonal damage in rats with SAH.

### OxyHb-induced axonal damage by promoting TDP-43 accumulation in the cytoplasm

**Fig. 3:**
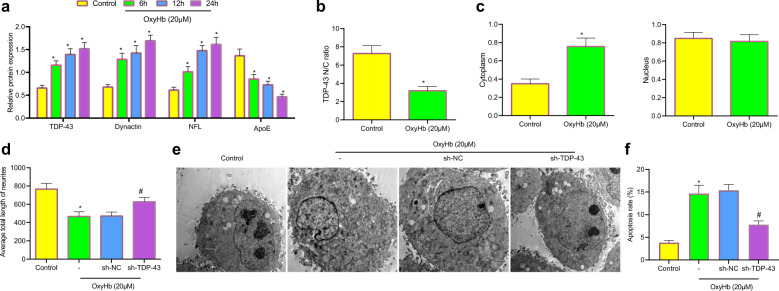
OxyHb induces neuronal and axonal damage, nuclear membrane abnormalities, and cell apoptosis by promoting the cytoplasmic accumulation of TDP-43. **a** TDP-43, dynactin, NFL and ApoE expression levels in neurons treated with OxyHb for 6, 12, and 24 h were determined by western blot analysis. **b** TDP-43 localization in cells was examined by immunofluorescence staining. **c** The expression of TDP-43 in the nucleus and cytoplasm was determined by nuclear and cytoplasmic fractionation experiments. **d** The colocalization of NAA and EGFP in cells was examined by immunofluorescence staining. **e** Damage to the nuclear membrane of neurons observed by TEM. **f** Apoptosis of neurons determined by flow cytometry. *P < 0.05 compared with control neurons, ^#^*P* < 0.05 compared with cells infected with sh-NC. The experiment was repeated three times independently.

To explore the underlying mechanism by which TDP-43 affects axonal damage, neurons were treated with 20 μM OxyHb for 6, 12, and 24 h. We showed that the expression of TDP-43 increased during the treatment time (Fig. 3a). The expression of axon damage marker proteins, including dynactin, neurofilament light (NFL) and apolipoprotein E (ApoE), was then measured, the protein expression of dynactin and NFL increased, while the level of ApoE decreased increasing OxyHb treatment time (Fig. 3a), indicating that OxyHb induced axonal damage. The position of TDP-43 in OxyHb-treated neurons was further observed by immunofluorescence staining, and the results showed that the localization of TDP-43 in the cytoplasm was notably increased (Fig. 3b). Nuclear and cytoplasmic separation experiments showed that the expression of TDP-43 in the cytoplasm increased notably (Fig. 3c). The length of neurofilament-associated antigen (NAA) antibody-labeled neurites and enhanced green fluorescent protein (EGFP)-labeled neurites was measured by immunofluorescence staining, and the results demonstrated that OxyHb decreased total neurite length in neurons, while TDP-43 silencing reversed this outcome (Fig. 3d). In addition, OxyHb treatment induced irregular nuclear morphology and invaginations of the nuclear membrane, which was reversed by TDP-43 silencing (Fig. 3e). Flow cytometry showed that neuronal apoptosis was increased with OxyHb treatment time, and TDP-43 silencing inhibited OxyHb-induced neuronal apoptosis (Fig. 3f). In summary, OxyHb induced cytoplasmic accumulation of TDP-43 and resulted in axonal damage and abnormal nuclear membrane morphology, and further promoted neuronal apoptosis. TDP-43 silencing alleviated axonal damage and abnormal nuclear membrane morphology and inhibited neuronal apoptosis.

### HDAC1/HSP70/TDP-43 triple complexes promoted cytoplasmic accumulation of TDP-43

**Fig. 4:**
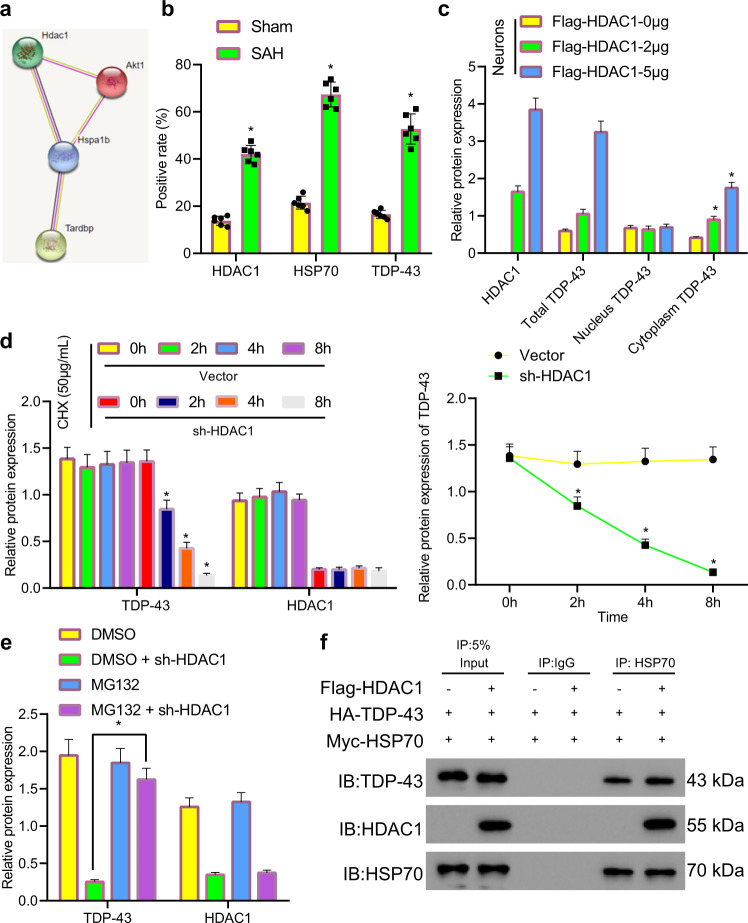
HDAC1 binds to HSP70 and TDP-43 to promote the cytoplasmic accumulation of TDP-43. **a** HDAC1/HSP70/TDP-43 interaction network predicted by the STRING website. **b** The colocalization of HDAC1 and TDP-43 as well as HSP70 and TDP-43 in brain tissues was examined by immunofluorescence staining. **c** The accumulation of TDP-43 in the cytoplasm was examined by nuclear and cytoplasmic separation experiments. **d** TDP-43 expression in sh-HDAC1-infected neurons after treatment with 50 μg/mL CHX was determined by western blot analysis. **e** TDP-43 expression in neurons after treatment with different concentrations of MG132 was determined by western blot analysis. **f** The binding of HDAC1, TDP-43, and HDAC1 in 293T cells was examined by Co-IP experiments. *P < 0.05 compared with the neurons of rats treated with sham operation/Vector/HDAC1-0 μg/DMSO + sh-HDAC1 *n* = 6. The cell experiment was repeated three times independently.

The STRING website predicted that HDAC1-HSP70 (Hspa1b)-TARDBP (TDP-43) was a regulatory pathway (Fig. 4a). The colocalization of HDAC1 and HSP70 with TDP-43 was examined, and the results demonstrated that the positive rate of HDAC1, HSP70, and TDP-43 in the brain tissues of rats with SAH increased notably compared with that in sham-operated rats, and the pathological colocalization resulted in the formation of point aggregates (Fig. 4b). Then, 293T cells were transfected with HDAC1 or HSP70. the Co-IP results showed that acetyl-K levels were sharply reduced (Supplementary Fig. 2a), indicating that HDAC1 mediated the deacetylation of HSP70. In addition, HDAC1 silencing notably enhanced HSP70 acetylation (Supplementary Fig. 2b). The expression of TDP-43 in the cytoplasm gradually increased with increasing HDAC1 concentrations, while TDP-43 expression in the nucleus remained unchanged (Fig. 4c). sh-HDAC1-treated neurons were treated with 50 μg/mL cycloheximide (CHX) for 0 h, 2 h, 4 h, and 8 h. TDP-43 protein levels gradually decreased with prolonged CHX treatment time (Fig. 4d). sh-HDAC1-treated neurons were treated with 5 μM of the proteasome inhibitor MG132. Western blot analysis revealed that MG132 inhibited the degradation of TDP-43 compared with that in neurons that were not treated with MG132 (Fig. 4e), indicating that HDAC1 silencing promoted TDP-43 degradation through the proteasome pathway. 293 T cells were transfected with Flag-HDAC1, HA-TDP-43, and Myc-HDAC1, and the Co-IP results revealed that these factors could bind with each other (Fig. 4f). In summary, HDAC1 could bind to HSP70 and TDP-43, promote HSP70 deacetylation and enhance TDP-43 accumulation in the cytoplasm while inhibiting protein degradation. In contrast, HDAC1 silencing promoted proteasomal degradation of TDP-43.

### HDAC1 silencing inhibited TDP-43 expression to reduce OxyHb-induced axonal damage

**Fig. 5:**
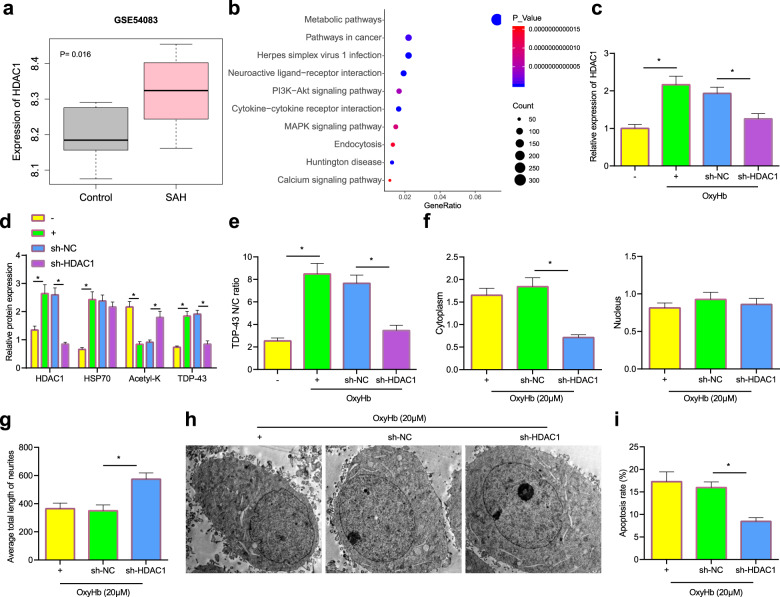
HDAC1 silencing reduces the formation of TDP-43 inclusions to alleviate axonal damage. **a** The level of HDAC1 in the GSE54083 microarray. **b** KEGG enrichment analysis of HDAC1-related genes. **c** The level of HDAC1 mRNA in neurons was measured by RT–qPCR. **d** The levels of HDAC1, HSP70, TDP-43, and acetyl-K in neurons were measured by western blot analysis. **e** The accumulation of HDAC1 and TDP-43 in the cytoplasm of neurons was determined by immunofluorescence staining. **f** The expression of TDP-43 in neurons was determined by nuclear and cytoplasmic fractionation experiments. **g** Neurite length was measured by immunofluorescence staining. **h** Nuclear membrane morphology was observed by TEM. **i** Apoptosis in neurons was determined by flow cytometry *P < 0.05. The cell experiment was repeated three times independently.

The SAH-related microarray GSE54083 was obtained from the GEO database and further analyzed, and the results revealed that HDAC1 was highly expressed in SAH (Fig. 5a). KEGG enrichment analysis showed that HDAC1-related genes were mainly enriched in pathways such as “Metabolic pathways”, “Neuroactive ligand–receptor interaction”, “Cytokine–cytokine receptor interaction”, “Herpes simplex virus 1 infection” and “Huntington disease” (Fig. 5b). RT–qPCR showed that OxyHb notably elevated the expression of HDAC1 in neurons, and sh-HDAC1 reduced the mRNA levels of HDAC1 in OxyHb-treated neurons (Fig. 5c). Based on the western blot results, OxyHb treatment promoted the expression of HDAC1 and TDP-43 in neurons and inhibited the level of acetyl-K, while sh-HDAC1 treatment reduced the levels of HDAC1 and TDP-43 but increased the acetyl-K level in OxyHb-treated neurons (Fig. 5d). Immunofluorescence staining indicated that OxyHb increased the level of TDP-43 in the cytoplasm, while sh-HDAC1 resulted in decreased TDP-43 in the cytoplasm (Fig. 5e). Moreover, we found reduced expression of TDP-43 in the cytoplasm but increased neurite length in neurons that were treated with OxyHb + sh-HDAC1 compared with those treated with OxyHb + sh-NC (Fig. 5f, g). TEM revealed that compared with that of neurons treated with OxyHb + sh-NC, the morphology of neurons treated with OxyHb + sh-HDAC1 was round and regular, and nuclear membrane morphology was normal (Fig. 5h). Flow cytometry showed that compared with neurons treated with OxyHb + sh-NC, neurons treated with OxyHb + sh-HDAC1 exhibited reduced apoptosis (Fig. 5i). In summary, HDAC1 silencing inhibited TDP-43 expression and alleviated OxyHb-induced axonal damage.

### SAHA alleviated neuronal damage by promoting TDP-43 degradation by maintaining the acetylation level of HDAC1/HSP70 complexes

**Fig. 6:**
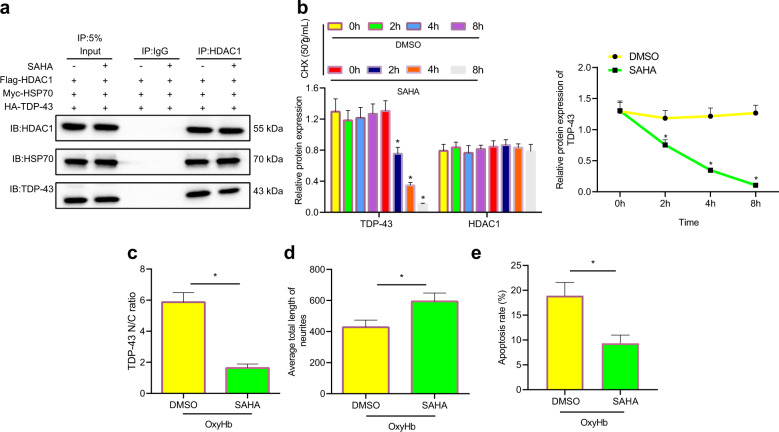
SAHA relieves axonal damage by enhancing the degradation of TDP-43 inclusions through HDAC1/HSP70. **a** The interaction of HDAC1, HSP70, and TDP-43 in 293T cells and neurons after SAHA induction was examined by Co-IP experiments. **b** The degradation of TDP-43 in CHX-treated neurons after SAHA treatment was examined by western blot analysis. **c** The colocalization of TDP-43 and HDAC1 in OxyHb-treated neurons after SAHA treatment was examined by immunofluorescence staining. **d** The colocalization of EGFP and NAA in cells was examined by immunofluorescence staining. **e** Apoptosis in cells after different transfections was determined by flow cytometry *P < 0.05. The cell experiment was repeated three times independently.

Neurons were treated with the HDAC1 inhibitor SAHA (1 μM) for 30 min, and the acetylation level of HSP70 was measured. SAHA treatment notably promoted HSP70 acetylation in 293T cells (Supplementary Fig. 3a). According to the IP results, a notable increase in HSP70 levels was observed (Supplementary Fig. 3b). SAHA-treated 293 T cells were further transfected with Flag-HDAC1, Myc-HSP70, and HA-TDP-43. The Co-IP results showed that the level of TDP-43 that immunoprecipitated with HDAC1 was markedly reduced (Fig. 6a), and the same results were observed in SAHA-treated neurons (Fig. 6a). CHX-treated neurons were further treated with SAHA, and the results showed that SAHA treatment markedly promoted the protein degradation of TDP-43 in neurons that were treated with CHX (Fig. 6b). The colocalization of TDP-43 and HDAC1 was examined by immunofluorescence staining, and the results showed that the accumulation of TDP-43 in the cytoplasm of neurons induced by OxyHb was reduced by SAHA treatment, and the ratio of nuclear-to-cytoplasmic TDP-43 was reduced (Fig. 6c). In addition, compared with that of neurons treated with OxyHb + DMSO, neurite length was increased by TDP-43 (Fig. 6d). Flow cytometry showed that compared with neurons treated with OxyHb + DMSO, neurons treated with OxyHb + SAHA had notably reduced levels of apoptosis (Fig. 6e). Overall, SAHA, which is an HDAC1 inhibitor, promoted the degradation of the TDP-43 protein by maintaining the acetylation of HSP70 and inhibiting the accumulation of TDP in the cytoplasm, ultimately alleviating neurite damage.

### SAHA relieved axonal damage and neurological dysfunction after SAH by inhibiting the HDAC1/HSP70/TDP-43 axis

**Fig. 7:**
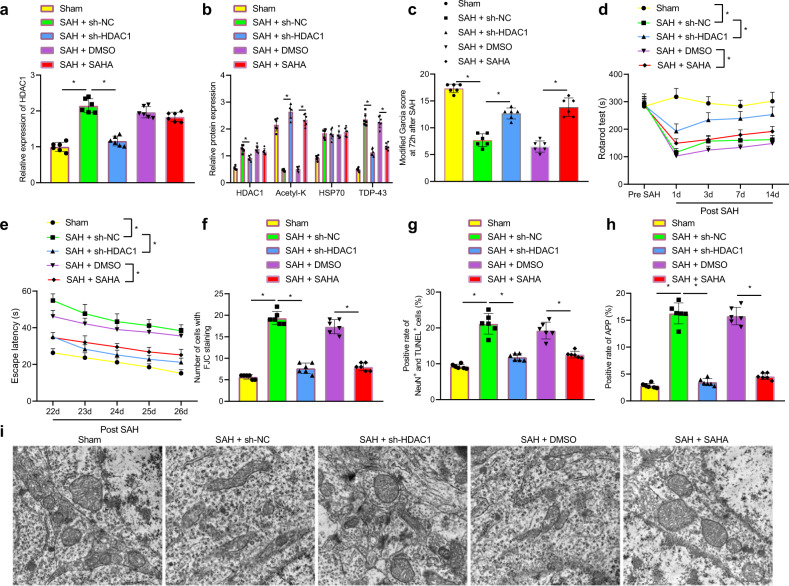
SAHA alleviates brain damage in SAH rats by suppressing HSP70/HDAC1/TDP-43. **a** HDAC1 mRNA levels in rats after different treatments were determined by RT–qPCR. **b** Protein levels of HDAC1, acetyl-K, HSP70, and TDP-43 in rats with SAH after different treatments was determined by western blot analysis. **c** Nerve damage in rats with SAH after different treatments was determined by the modified Garcia behavior score. **d** The movement of rats with SAH after different treatments was determined by the rotarod test. **e** Memory impairment in rats with SAH after different treatments was determined by the Morris water maze test. **f** The degeneration of cortical neurons in rats with SAH after different treatments was determined by FJC staining. **g** Apoptosis in neurons was determined by TUNEL and NeuN double staining. **h** APP expression in rats with SAH after different treatments was determined by IHC staining. **i** Axonal damage in the brain tissue of rats with SAH after different treatments was observed by TEM. *P < 0.05, *n* = 6. The experiment was repeated three times independently.

To further explore whether the deacetylase inhibitor SAHA is involved in the regulation of nerve damage in SAH by mediating the HDAC1/HSP70/TDP-43 axis, SAH model rats were injected with lentivirus-mediated sh-HDAC1 and SAHA in the lateral ventricle. RT–qPCR analysis showed that the level of HDAC1 in rats with SAH that were injected with sh-NC was notably increased relative to that in sham-operated rats. The level of HDAC1 was notably decreased in rats with SAH that were injected with sh-HDAC1 compared with that of rats with SAH that were injected with sh-NC. Compared with that of rats with SAH that were treated with DMSO, the level of HDAC1 showed no obvious change in rats with SAH that were treated with SAHA (Fig. 7a). In addition, the levels of HDAC1 and TDP-43 in rats with SAH that were injected with sh-HDAC1 were reduced, the level of acetyl-K was elevated, and the level of HSP70 remained unchanged relative to the effect of sh-NC treatment. Compared with rats with SAH that were treated with DMSO, acetyl-K levels increased markedly, HDAC1 and HSP70 levels remained unchanged, and TDP-43 levels decreased notably in rats with SAH that were treated with SAHA (Fig. 7b). Cognitive tests showed that compared with that of rats with SAH that were injected with sh-NC, the neurological damage score was reduced, and the exercise capacity and memory level were increased in rats with SAH that were injected with sh-HDAC1; the same behavioral phenotype was observed after SAHA treatment (Fig. 7c–e). Then, FJC and TUNEL staining demonstrated that in comparison to that of rats with SAH that were injected with sh-NC, the number of FJC-positive cells and the number of NeuN^+^TUNEL^+^ cells in rats with SAH that were injected with sh-HDAC1 were reduced, and the same trend was observed after SAHA treatment (Fig. 7f, g), indicating that neuronal damage was reduced. APP expression was then examined by IHC staining, and the results demonstrated that the number of APP-positive cells in rats with SAH that were injected with sh-HDAC1 was largely decreased compared with that of rats with SAH that were injected with sh-NC, and SAHA treatment inhibited the positive rate of APP in brain tissues (Fig. 7h). The number of swollen and dystrophic axons in rats with SAH that were injected with sh-NC was notably reduced compared with that in rats with SAH that were injected with sh-HDAC1, and SAHA treatment alleviated axonal damage (Fig. 7i). In conclusion, SAHA alleviated axonal damage and cognitive impairment by mediating the degradation of TDP-43 through the acetylation of HSP70 and the inhibition of HDAC1.

## Discussion

The average SAH patient is 55 years old, and this condition commonly leads to cognitive impairments in survivors because of axonal damage and white matter injury^24,25^. Much attention has been given to the function of various HDAC inhibitors in the progression of intracerebral hemorrhage, including SAH^26,27^. Here, we attempted to clarify the mechanisms of the HDAC inhibitor SAHA in SAH, and our results revealed that SAHA relieved neuronal and axonal damage by promoting TDP-43 degradation by enhancing the acetylation of HSP70 through HDAC1 inhibition, thereby attenuating the development of SAH.

We discovered that TDP-43 was highly expressed in the cerebrospinal fluid of SAH patients and in the brain tissues of a rat model of SAH. Consistent with our findings, a previous study also showed upregulated expression of TDP-43 in the cerebrospinal fluid of patients with aneurysmal SAH and the brain tissues of a rat model of SAH^17^. Furthermore, our study revealed that TDP-43 overexpression enhanced cognitive impairment in rats with SAH in vivo and that TDP-43 accumulation in the cytoplasm in a neuronal cell model of SAH worsened axonal damage in vitro. Cytoplasmic mislocalization is a characteristic of TDP-43 pathology and is further recognized as a hallmark of neurodegenerative diseases such as amyotrophic lateral sclerosis (ALS), frontotemporal lobar degeneration (FTLD), Parkinson’s disease and Huntington’s disease^28–30^. Specifically, TDP-43 acetylation increased the accumulation of insoluble and hyperphosphorylated TDP-43 inclusions, which contributed to the pathogenesis of ALS and FTLD^31^. Another study showed that TDP-43 promoted Alzheimer’s disease development^32^. In summary, these lines of evidence validated the exacerbating role of TDP-43 in SAH progression.

Then, TDP-43 protein degradation was inhibited by HSP70 deacetylation, which induced cytoplasmic aggregation and further promoted inclusion body formation. Similar to our findings, another study showed that the pathological accumulation of insoluble TDP-43 could be reduced by HSPs and attenuate amyotrophic lateral sclerosis^33^. Moreover, the deacetylation of HSP70 was promoted by HDAC1, and HDAC1 silencing promoted TDP-43 degradation, thereby alleviating neuronal and axonal damage, abnormal nuclear membrane morphology, and apoptosis in a neuronal cell model of SAH. A prior study showed that protein deacetylation, which is the reverse process of acetylation (a posttranslational modification), was mediated by deacetylases^34^. Moreover, the inhibition of HDACs was reported to exert a protective effect against acute lung injury by acetylating and suppressing HSP90 activity^35^. Moreover, HDAC silencing was shown to reduce white matter damage after intracerebral hemorrhage^36^. Furthermore, this study showed that the HDAC inhibitor SAHA alleviated axonal injury and cognitive impairment in rats with SAH by elevating TDP-43 degradation by enhancing the acetylation of HSP70 by inhibiting HDAC1 enzymatic activity. SAHA is a well-recognized HDAC inhibitor that has also been confirmed to alleviate intracerebral hemorrhage^9^ In addition, a recent study revealed that SAHA attenuated hemorrhagic shock and resuscitation-induced lung injury by repressing histone acetylation^37^. Hence, these findings support the role of SAHA in ameliorating SAH development by regulating the HDAC1/HSP70/TDP-43 axis.

**Fig. 8:**
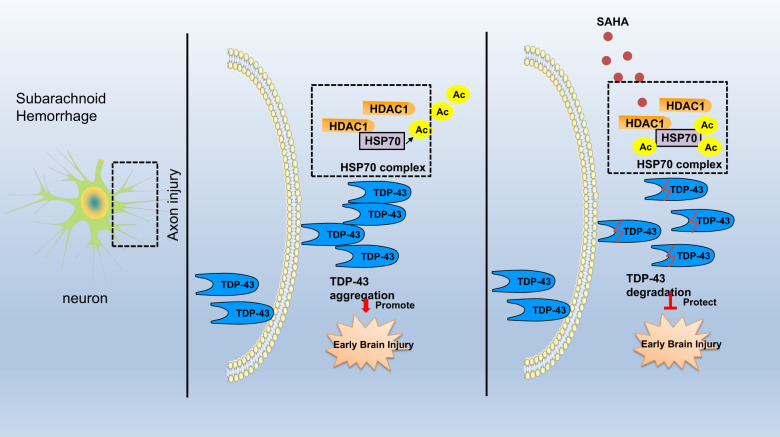
The molecular mechanism by which SAHA alleviates early SAH. SAH induces HDAC1 binding to HSP70 and promotes TDP-43 accumulation in the cytoplasm to form inclusion bodies, ultimately promoting neuronal and axonal damage and inducing brain damage in early SAH. SAHA protects against early SAH by promoting the degradation of TDP-43 by enhancing HSP70 acetylation through the inhibition of HDAC1.

Taken together, the findings of this study confirmed that SAHA plays an inhibitory role in SAH progression by promoting the degradation of TDP-43 by enhancing the acetylation of HSP through the suppression of HDAC1 activity (Fig. 8). However, the efficacy of HDAC inhibitors in SAH is a novel research focus that needs further investigation.

## Supplementary information

Supplementary information
