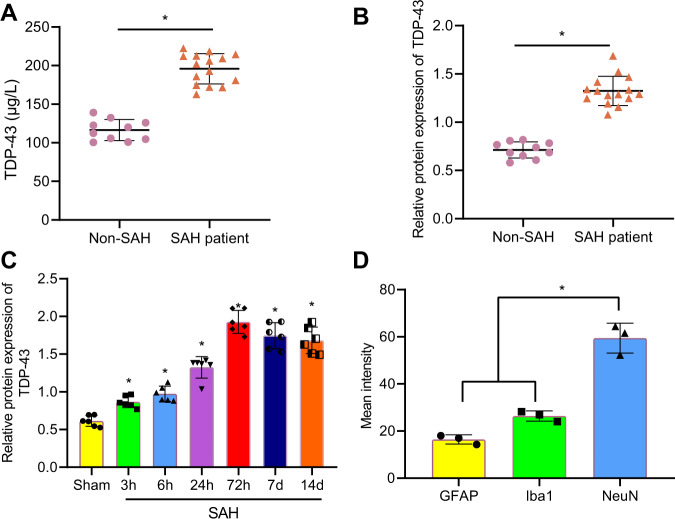# Correction: Suberoylanilide hydroxamic acid suppresses axonal damage and neurological dysfunction after subarachnoid hemorrhage via the HDAC1/HSP70/TDP-43 axis

**DOI:** 10.1038/s12276-022-00783-3

**Published:** 2022-07-04

**Authors:** Kui Luo, Zhifei Wang, Kai Zhuang, Shishan Yuan, Fei Liu, Aihua Liu

**Affiliations:** 1 Department of Neurosurgery, The Third Xiangya Hospital, Central South University, 410013 Changsha, China; 2 Medical College, Hunan Normal University, 410000 Changsha, China; 3 Department of Neurosurgery, The Fifth Affiliated Hospital of Sun Yat-Sen University, 519000 Zhuhai, China; 4 Beijing Neurosurgical Institute, Beijing Tiantan Hospital, Capital Medical University, 100070 Beijing, China

**Keywords:** Neuroscience, Molecular biology

Correction to: *Experimental & Molecular Medicine* 10.1038/s12276-022-00761-9, published online 02 May 2022

In this article the unit in the Vertical Axis in figure 1A should be corrected; the figure should have appeared as shown below.

The original article has been corrected.