# Supplementary material for: Suberoylanilide hydroxamic acid suppresses axonal damage and neurological dysfunction after subarachnoid hemorrhage via the HDAC1/HSP70/TDP-43 axis
Source: Exp Mol Med. 2022 May 2;54(9):1423–33. doi: 10.1038/s12276-022-00761-9 (PMC9535006; doi:10.1038/s12276-022-00761-9)
Supplement: Supplementary file 1 — Supplementary information [file 12276_2022_761_MOESM1_ESM.pdf]

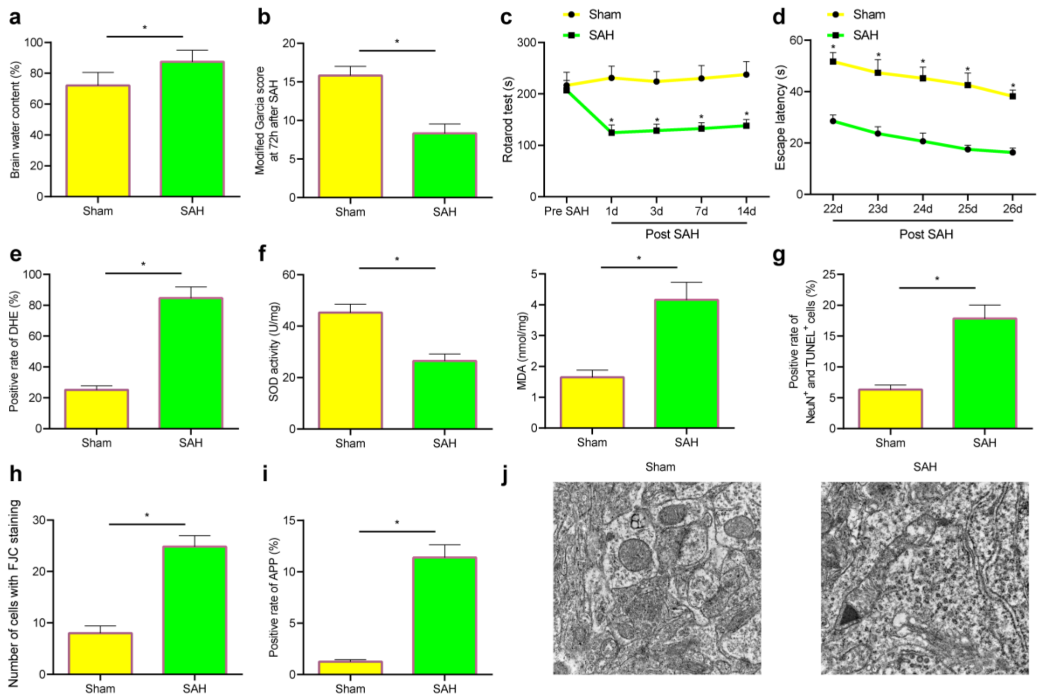

Supplementary Fig. 1 Nerve function and axonal damage in rat model of SAH. a, The water content of brain tissues determined by dry/wet method. b, Nerve damage evaluated by Modified Garcia behavior score. c, Movement of rats 1 d before and 3 d, 7 d, 14 d after SAH modeling detected by rotarod experiment. d, Memory function of rats 22 d, 23 d, 24 d, 25 d, 26 d after SAH modeling detected by Morris water maze test. e, The active oxygen in the brain tissues of rats 72 h after SAH modeling determined by DHE staining. f, SOD activity and MDA content 72 h after SAH. g, Apoptosis of neurons 72 h after SAH determined by TUNEL and NeuN double staining. h, Neuronal cell degeneration 72 h after SAH modeling determined by FJC staining. i, APP expression at 72 h after SAH modeling determined by IHC staining. j, Electron microscopic images of cerebral infarction (left and middle panels) and hairy stem (right panel) after subarachnoid hemorrhage. * p < 0.05, n = 6. The experiment was repeated 3 times independently.

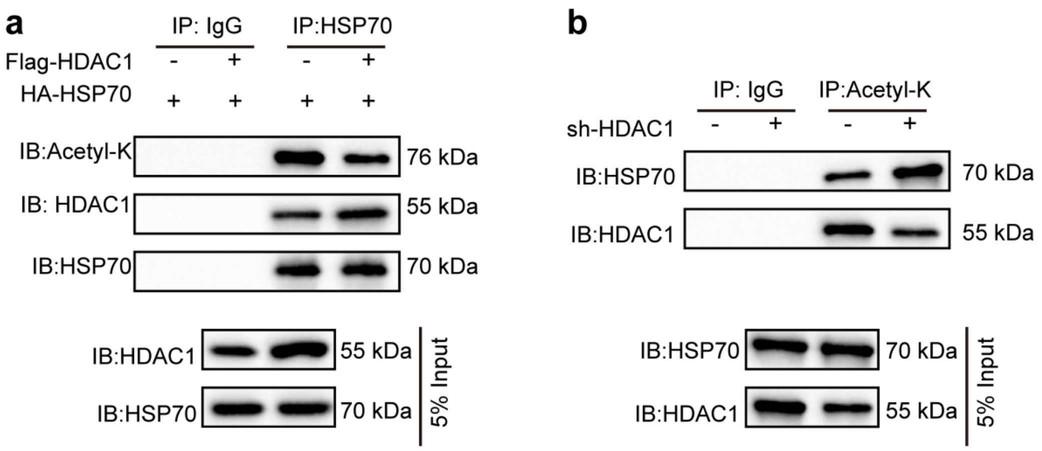

Supplementary Fig. 2 Deacetylation modification of HDAC1 to HSP70. a, The level of Acetyl-K in 293T cells transfected with Flag-HDAC1 or HA-HSP70 after the treatment of MG-132 determined by Co-IP experiment. b, HSP70 acetylation in sh-HDAC1-treated neurons treated by MG132 determined by Co-IP experiment. The experiment was repeated three times independently.

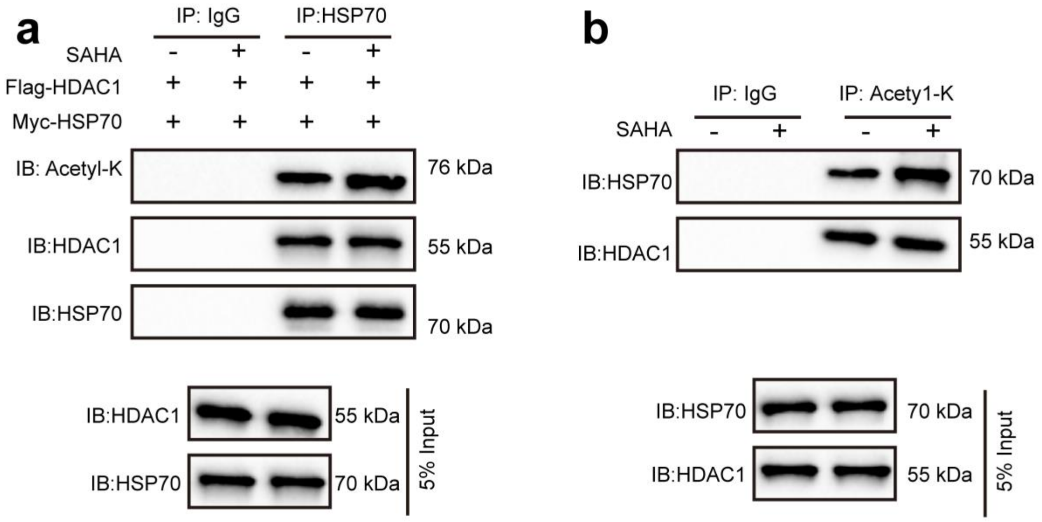

Supplementary Fig. 3 The effect of SAHA on acetylation level of HSP70 through HDAC1. a, HSP70 acetylation level in SAHA-induced 293T cells transfected with Flag-HDAC1 or Myc-HSP70 after MG132 treatment determined by Co-IP experiment. b, Acetyl-K levels in SAHA-induced neurons after MG-132 treatment determined by Co-IP experiment. The experiment was repeated three times independently.

Supplementary Table 1 Primer sequence for RT-qPCR

Genes	Species	Primer sequence
TDP-43	Rno	F: 5'-TGCGAACATGGACTGAGCTT-3'
		R: 5'-GCATTCAGGGCTGCAAAACA-3'
HDAC1		F: 5'-ACTAGATAGGGACCAGCGCA-3'
		R: 5'-AGCTCCTAAGCAGGCACTTG-3'
GAPDH		F: 5'-GACATCAAGAAGGTGGTGAA-3'
		R: 5'-TGTCATACCAGGAAATGAGC-3'

Note: TDP-43, transactive response DNA-binding protein 43; HDAC1, histone deacetylase 1; GAPDH, glyceraldehyde-3-phosphate dehydrogenase; RT-qPCR, reverse transcription-quantitative polymerase chain reaction; F, forward; R, reversed.
